# Education research is still the hardest science: a proposal for improving its trustworthiness and usability

**DOI:** 10.12688/f1000research.109700.1

**Published:** 2022-02-24

**Authors:** Gustavo Fischman, Audrey Amrein-Beardsley, Stephanie McBride-Schreiner

**Affiliations:** 1 Mary Lou Fulton Teachers College, Arizona State University, Tempe, AZ, 85287-1611, USA

**Keywords:** colleges of education; research impact; research assessment

## Abstract

In this essay, we argue that colleges of education, particularly those at research-intensive institutions, favor simplistic notions of scholarly impact and that this trend has concerning implications for the field, for researchers, and for the public at large. After describing the challenges and shortcomings of the current models of research assessment in education, we outline an alternative proposal in which trustworthiness and usability of research would complement traditional metrics of scholarly relevance. This proposal encourages a twofold approach to research assessment that involves (1) a more thorough analysis of the limitations and problems generated by the use of simplistic notions of scholarly impact, and (2) a commitment to the implementation of more equitable systems based on a broader range of assessment measures to assess faculty research contributions.

## Introduction

Two decades ago, David C. Berliner (2002) warned of the risks involved when research funding policies are based on narrow definitions of what acceptable science is. He argued that:

“Hard-to-do science is what the social scientists do and, in particular, it is what we educational researchers do. In my estimation, we have the hardest-to-do science of them all! We do our science under conditions that physical scientists find intolerable. We face particular problems and must deal with local conditions that limit generalizations and theory building—problems that are different from those faced by the easier-to-do sciences.” (p. 18)

Today, government funding agencies still give preference in education to “easier-to-do sciences” when it comes to research methods. The good news, however, is that they no longer explicitly exclude multiple approaches for conducting research in education. The bad news is that the field of education research itself has adopted a very narrow set of indicators for judging what is acceptable as good research.

As faculty, researchers, scholars, and editors working in higher education, we see an unsettling trend. Measures of impact—social, global, and real-world—are increasingly expected of scholarly research; yet, the assessment of these outcomes remains vague, arbitrary, and one-size-fits-all across disciplines. Further, the scholarly publishing ecosystem, which produces the most revered and measurable indicators of scholarly impact and innovation, has grown more commercialized and profit driven, leading to an ever wider disconnect between the producers of scholarly knowledge ( *e.g.*, researchers, funding agencies, and the community at large) and the conveyors of that knowledge ( *e.g.*, academic publishers). We argue that those in higher education in general, but colleges of education in particular, need to discuss and challenge simplistic notions of scholarly impact and move towards a more trustworthy and informed culture and infrastructure of scholarly assessment.

A common understanding of scholarly impact is often related to two indicators: number of articles in flagship journals and number of citations ( Aguinis *et al.*, 2012). For many scholars and organizations ( Anderson *et al*., 2021; Hicks *et al*., 2015; Walker, 2017), however, such definitions of impact, especially when institutionalized in college standards or university tenure and promotion policies and procedures, are not only inadequate to assess scholarly relevance, but also embody the wrong kind of incentives.

Consider the following scenario. A professor with research expertise in high-interest topics writes an insightful blog with thousands of non-academic followers. When evaluated for promotion, the professor is told by senior faculty *not* to mention the blog given its lesser academic status, despite the fact that education influencers like Diane Ravitch, Frederick Hess, and Mercedes Schneider have enormous blog audiences with millions of page views each.

Take another example. A professor, who is frequently consulted by research organizations in other countries about implementing good practices in scholarly publications and the organization of academic events is advised that for promotion purposes only collaborations with reputable United States (U.S.)-based organizations will be considered. How can such activities, which clearly build on these professors’ research and expertise, increase the recognition and prestige of the professors and their affiliated institutions, disseminate relevant knowledge without barriers, and offer evidence of significant reach and contribution to the public good, be viewed as so marginal in terms of scholarly impact?

Another often overlooked and misunderstood dimension of the assessment of scholarly impact is the structure and culture of scholarly publishing. For example, a journal might be consulted about its impact to help decide a tenure and promotion case at a university. All members but one on the review panel recommend tenure. The hold-out states that the author did not publish in enough high impact journals. One of the journals in question is an open access (OA) publication recognized as influential by many scientific organizations, with thousands of academic and non-academic readers worldwide and a solid record of citations per article, but due to its nontraditional publishing structure and multilingual nature, it was denied a journal impact factor by the publishing affiliate that assigns such measures. How could such a journal be excluded from recognized (albeit imperfect) indicators of impact and then, because of that peculiarity, be branded as *not* high impact when article-level and engagement metrics suggest otherwise?

Here we propose that the field of education research moves away from the imperfect and ineffective notion of impact and similar terms like “returns”, “benefits” and “value”, and toward more comprehensive and field-specific scholarly assessment strategies. In the next section, we focus on the challenges and shortcomings of the current models of assessment of research impact in education, and then outline a proposal in which the trustworthiness and usability of research would complement current metrics of scholarly relevance.

## Is *impact* a new fetish of education research?

In the U.S. and globally, colleges of education, primarily at research intensive universities are converging on the idea that evidence of impact is of utmost importance. Impact has become a new fetish ( Wood, 2021). This increased fascination with finding better indicators of scholarly impact and influence relies on formulaic uses of metrics-based reward and punishment assessment processes to accomplish three simultaneous and elusive goals: increase research impact, enhance institutional prestige, and demonstrate high levels of scholarly productivity and innovation ( Schneider, 2015).

Today it is rare to find colleges of education that are *not* requesting faculty to provide annual evaluative reports with measurable metrics, such as numbers of articles published in “High Impact Factor Journals,” numbers of citations per article, and other indicators of *impact* ( *e.g.*, Google Scholar’s *h-indices*, Publish or Perish scores, levels of engagement in Kudos, the RG Score from Research Gate). Also pertinent in judging the *quality* of a faculty member’s work are indicators such as publications in journals with high rejection rates or sponsored by esteemed professional associations ( *e.g.*, American Educational Research Association [AERA]; publications by university presses ( *e.g.*, Oxford University Press); and funds, grants, and research awards bestowed by organizations ( *e.g.*, Institute of Education Sciences [IES]).

This model of holding university professors accountable for their presumed impact, or rather the impact of their scholarly products and dossiers, is not new ( Boyer, 1996; Weiss, 1981), nor exclusive to colleges of education, however. Many fields are subject to the “metric tide” ( Wilsdon et al., 2015) and “ranking mania” ( European University Association, 2013, p. 6) that increasingly frames the assessment and evaluation policies and procedures of contemporary universities. As Shewchuk and Cooper (2018) concluded, after conducting an analysis of 721 indicators of research impact for social sciences in 32 countries:

“What is clear from the veritable explosion of research impact materials in the past decade and the increasing number of performance-based research funding systems arising globally is that research impact will be a defining factor of research infrastructure, funding and landscapes across the world for the foreseeable future.” (p. 63)

We do not oppose the use of clear indicators and metrics to assess research and defend the principle of scholars’ curiosity as driver of scientific endeavors. We also do not support a nostalgic return to evaluation systems used during idealized eras of universities governed by autonomous communities of scholars. We do, however, believe education researchers need to be more cautious and identify, resist, and replace assessment policies based on poorly constructed and misleading metrics that will not improve education research, nor its usability, relevance, and value.

## Can we assess scholarly contributions in education without being simplistic?

What is research impact? There is a distinction between *academic impact*, understood as the intellectual contribution to one’s field of study within academia, and *external socio-economic impact,* effects beyond academia ( Penfield *et al*., 2014). *Impact* is multifaceted, dynamic, temporal, and not always beneficial. Meanings and judgments of impact differ across disciplines and vary as cultures, policies, and contexts change.

Few would dispute the claim that the *impact* of education research is both elusive and subjective. As Kaestle (1993) noted in *The Awful Reputation of Education Research,* the goal to increase the reputation and impact of educational scholarship has deep roots: “[I] f education researchers could reverse their reputation for irrelevance, politicization, and disarray […] they could rely on better support because most people, in the government and the public at large, believe that education is critically important” (pp. 30–31).

Some educational researchers have attempted to collaborate with practitioners to yield more impactful research to address problems in real classrooms, schools, and universities ( Penuel *et al.*, 2016). Yet, as Berliner (2002) underscored in the opening quote, due to the varied and complex nature of education systems, education research is contextual. Types of research also matter. Current impact indictors favor research with more immediate visible results over other types of research with less immediate or tangible impact ( Laing *et al*., 2018).

Colleges of education, then, face a conundrum. Despite ample consensus regarding the desirability of producing more studies with the explicit purpose of improving education ( Penuel *et al*., 2016), and more broadly focused research oriented to the public good, no effective and fair system exists that captures the full picture of the relevance and impact of scholarship in a field as diverse as education ( Anderson *et al*., 2021; Simons, 2008).

Consequently, in most cases instead of adopting contextualized and measured models, many colleges of education adopt overly simplified systems of impact assessment based on indirect measures of scholarly relevance such as the Journal Citation Record (JCR) from Scopus, and the Journal Impact Factor (JIF), published Web of Science. These measures have been long been controversial ( Alperin *et al.*, 2019; Simons, 2008) yet convey a sense of being purely meritocratic, by using appropriate indicators of impact, relevance, and influence ( Fischman, 2016; Zuiker *et al*., 2019). In other words, the indicators easiest to quantify may or may not promote the most impactful education research. Such metrics are developed using sophisticated algorithms that yield robust statistics to be consumed and trusted, and also ranked, categorized, monetized, regardless of the validity of the inferences derived. This phenomenon, the “simplimetrification of educational research” ( Fischman, 2016), has the ironic effect of allowing researchers and their institutions to feel good about themselves, by confusing continuous increases of countable items ( *e.g.*, the more articles and more citations in more exclusive journals) with substantial scientific and pedagogical contributions.

## The academic publishing dimension

This metric tide, in conjunction with the *publish or perish* imperative, has generated a veritable tsunami with gigantic waves of articles that follow pre-established, tidy paths of exploration that may be accurately measured and rewarded. The publication of educational research, however, is not completely altruistic or disinterested. Academic publishers and editors are keen to prove that the research they publish is influential in order to attract new submissions and subscriptions. To attract funding and prospective students, researchers and administrators are keen to prove that the research they produce is influential. Both the JCR and the JIF—assigned to journals, not individual articles—are attractive, recognizable metrics that conflate journal performance with individual researcher performance, thus serving the aforementioned, multiple interests simultaneously.

As Puehringer *et al*. (2021) noted, the political economy of academic publishing entails “publishers sell a highly profitable, yet immensely publicly subsidized product” (p. 2). Academic publishing is a vast, lucrative industry, with revenues estimated at USD 26 billion ( Johnson *et al*., 2018). The rising demand for free and digital access to research over the last 30 years led commercial publishers to adopt hybrid models that balance traditional practices ( *e.g.*, *via* subscriptions and paywalls) with new OA schemes that offset publishing costs by charging researcher-authors—often paid out of research accounts provided by institutions or funders—to make published content freely available. Further, academic libraries broker serial deals with commercial publishers to access the same content through subscription bundles, essentially buying back access to the research that the researchers at their institutions produced ( Wenzler, 2017). The ironic result is that much potentially impactful research is hidden away behind paywalls because many authors choose not to, or are unable to pay the fees to make their research available to all.

Linked to these financial aspects, scholarly publication formats (print, digital, hybrid) and types (subscription-based, OA, *etc.*) are complex. Further, multiple OA publishing options exist, including but not limited to *Gold OA, Delayed OA*, *Green OA*, and *Platinum or Diamond OA.* Adding to this complexity, OA articles have a range of copyright licenses with varying degrees of permissions. A lack of awareness among researchers about the differences between publication types and associated licensing leads to the general misconception that all OA publications are free, which is untrue. All publications have a cost; the difference is *who* pays (readers, authors, institutions, libraries, funders, publishers, *etc.*)

Inequities embedded in the scholarly publishing landscape, such as biases for English-language works ( *e.g.*, more than 33% scientific documents on global conservation are published in languages other than English but are critically ignored; see, *e.g.*, Amano, 2021), also have implications for research assessment that are frequently overlooked ( Kubota, 2020). The circularity of these biases, stemming from the Western market-oriented nature of scholarly publishing, are striking. For example, a journal article indexed in Scopus or Web of Science is viewed as an indicator of research quality and international reach ( Sivertsen, 2016, p. 357). Journals registered in these influential indexes are more likely to publish English-only articles, given the editorial boards and editors also conduct their activities in English ( Vasen & Vilchis, 2017). Moreover, a journal article published in U.S.-based Scopus or UK-based Web of Science is more likely to have a JIF, also assigned by Web of Science. In fields like the social sciences, journals with high JIFs tend to have higher APCs, potentially excluding submissions by authors from less affluent countries, who are not native English speakers, or both ( Demeter & Istratii, 2020, p. 506). Considering all of the above, in some subject areas, the correlation between high APCs and JIF and JCR, combined with the existing economic inequalities among countries, reinforces existing hierarchies of language as maintained by publisher databases.

While no scholarly or business enterprise is perfectly equitable, such biases and circularity—and their reinforcement through academic research assessment processes—are highly concerning. Commercial publishers capitalize on the decentralized, siloed nature of academic institutions and research communities (who are also in competition for research dollars and rankings) and a lack of in-depth knowledge about the scholarly publishing process. Researchers should be wary of giving up more control over who is defining and measuring research quality and impact ( Aspesi & Scholarly Publishing and Academic Resources Coalition [SPARC], 2019). Given that quality, levels of international engagement, and societal relevance certainly should be promoted in research assessment, should coverage by a commercial indexing service be a criterion for research quality or an indicator of global engagement?

## Resisting the metric tide in education scholarship: trustworthiness and usability

In our view, this model rewards people based on metrics and measurements that do not differentiate between research articles concluding with the statement “more research is needed” and those that bring value to a scholarly field, help educators improve their practice, or supply compelling evidence to policymakers for important decisions. Rather, education researchers learn new terms and tools about scholarly assessment, instead of expanding curious research, asking better and more relevant questions for the advancement of the field, or producing more usable knowledge. Unfortunately, this fascination with simpler models, combined with a disconnect from the publishing ecosystem, ultimately lead researchers to an uncritical and sometimes naïve acceptance of the accuracy and explanatory power of these indicators.

In recent years, a number of initiatives have emerged to push against this tide of simplimetrification, as groups of researchers have converged to develop guidelines for research evaluation and assessment without using one-dimensional measures. Some prominent examples include the Leiden Manifesto ( http://www.leidenmanifesto.org/), the San Francisco Declaration on Research Assessment (DORA; https://sfdora.org/), the Panama Declaration of Open Science ( https://web.karisma.org.co/declaraciondepanama/), and the Hong Kong Principles ( https://www.wcrif.org/guidance/hong-kong-principles). Since 2019, the Hong Kong Principles, for example, have promoted research assessment based on five key tenets: responsible research practices, transparent reporting, open science (open research), valuing diverse types of research, and recognizing all contributions to scholarly activity. Collectively, these researcher-led activities represent pushback against an unbalanced system of research assessment in which individual researchers face a “one-sided emphasis on traditional, quantifiable output indicators,” despite the fact that “bibliometric indicators tell a story, but not the whole story” ( Dutch Research Council, 2019, p. 4).

Such recommendations are, accordingly, gaining traction, and advocates are moving words into action. In 2019, *Consejo Latinoamericano en Ciencias Sociales* (CLACSO; https://www.clacso.org/) organized FOLEC— *Foro Latinoamericano sobre Evaluación Científica* (Latin American Forum of Scientific Evaluation)—to develop better systems of assessment consistent with Open Science principles. The metric tide is turning in Asia, as shown by the Chinese government’s decision to stop using the JIF and similar indirect metrics as the key indicator of research assessment ( Zhang & Sivertsen, 2020). Also noteworthy, the European Research Council decided to disallow mention of indirect journal metrics in research funding applications ( Matthews, 2021). The call by Dutch universities and funding agencies for a revamped system of recognition and rewards, based on diverse talent, academic interdependence, emphases on quality over quantity, and the encouragement of open science and high-quality leadership, captures the essence of these initiatives ( Dutch Research Council, 2019, p. 3).

## Should trustworthiness and usability be considered in assessing scholarship in education?

Building on the heavy lifting of those mentioned in the previous section, we propose that a better way to assess education research requires a combined use of existing indicators and metrics with evidence of enhanced trustworthiness and usability—within and beyond disciplinary, professional, or technical communities—to foster and sustain processes of conceptual inquiry and education problem solving. As per the aforementioned Hong Kong Principles, “The primary goal of research is to advance knowledge. For that knowledge to benefit research and society, it must be trustworthy. Trustworthy research is robust, rigorous and transparent at all stages of design, execution and reporting” ( Moher *et al*., 2020, p.1).

Trustworthiness is not a given and not an eternal quality ( Schwandt *et al*., 2007). Robust findings may be trustworthy in one decade and not another. As the group Science in Transition ( Dijstelbloem *et al*., 2013) pointed out, researchers must also address the increasing mistrust from the public about *scientific expertise* and tell the public how science really works. Trust in the results of education research, no matter how rigorous the procedures used, is never simply assumed. Trusting the process and results of any research will always involve moral, cultural, and political considerations ( Little & Green, 2021). To increase *the trustworthiness* of education research, it is necessary, yet not sufficient, to provide wide access to the knowledge produced and engagement with the ideas and data derived from such research.

Access to and engagement with scholarship entail more than depositing knowledge in the library or an OA journal, book, or repository. These matters also rest on other aspects of research, such as language, previous knowledge of the phenomenon studied, ideological preferences, and the like ( Suber, 2016). Regarding *access*, how easy might it be to access the knowledge produced and what might be the barriers to accessing that knowledge? Did the knowledge reach its intended audiences ( *e.g.*, scholars, professionals, policy makers, or practitioners in the field)? Did the knowledge reach general, non-targeted audiences? Regarding *engagement*, to what extent do the central ideas, procedures, data, and conclusions enter into our systems of knowledge exchange with our intended audiences? In other words, if the research is not accessible due to various barriers ( *e.g.*, language, technology, disability, paywalls, and the like) then how can it be considered trustworthy by its intended audiences?

Education research will not be usable unless it is trusted; thus, trustworthiness and usability are inexorably linked. By usability we mean processes that signal potential access and engagement by both specialists as well as practitioners, each group accessing and engaging on their own terms, in their own time, and according to their own needs. This notion of usability also requires access and engagement with five critical components of knowledge generated by research: learnability, efficiency, memorability, integrity, and satisfaction derived via the knowledge produced ( Han *et al*., 2001).

In our understanding, usability is not a measure of dissemination or implementation, nor a description of processes or products. Here, we want to emphasize that we are not advocating for usefulness as a key indicator of relevance as others have done ( Buckhardt & Shoenfeld, 2003). We welcome research that has direct applications in teaching and learning. But defending the principle that practical and immediate implementation is not, and should not be, the goal of *all* education research. Conceptual studies directed at understanding and developing theories, for example, could prove very relevant and usable. What we propose, instead, is that at the institutional level education research should be promoted, and thus incentivized and assessed considering its *usability*, not only in the abstract form of the well-known questions of “So what?” and “Who cares?” but also in concrete steps taken to support strategies that help researchers mobilize research results.

The condition that we want to underscore is that trustworthiness and usability are not intrinsic qualities of the knowledge derived from any research endeavor, but characteristics that require intentional strategies that need to be incentivized to be implemented. We agree with others (viz., Berliner, 2002; Campbell et al., 2017; Hess, 2008) who warned about the shortcomings of reducing education research to methodological or technical matters. Improving access and engagement and opening diverse dialogues among researchers, policymakers, practitioners, and the public demand close attention to techniques and methods, but an even closer engagement with what is ethically, politically, and pedagogically desirable. As scholars, these desirable outcomes are linked to greater opportunities for open, interdisciplinary, and intersectional inquiries, welcoming a plurality of epistemic standpoints, and strengthening the commitment to contribute to the public good. Next, we ask what can be done to encourage more comprehensive assessments of education research.

## What can be done?

Perhaps the first step is to interrogate and challenge the notion that the simplimetrification of assessing research in education is unavoidable. Understanding its administrative advantages as systems of distributions of rewards and punishments, as well as acknowledging its shortcomings, is the first step toward more trustworthy and usable research in education.

If we reconsider the opening vignettes in light of a new model based on trustworthiness and usability, quite a different story of assessment unfolds. Professors writing a blog with thousands of primarily non-academic followers would be supported and recognized in their efforts to interact with the public and build trust in scholarship through blogging. Professors consulted by numerous international research organizations in other countries would be supported and recognized for their contributions to global engagement and support of multilingualism within scholarly communications, both of which foster trust in research between U.S.-based or non-U.S.-based research communities.

One potential path to an assessment system based on trust and usability is a renewed commitment to the *raison d'être* of education research: its pedagogical function. As the editors of the *British Journal of Educational Research* argued, the field needs to combine the search for identifying and posing education problems with inquiries that pose solutions to those same problems:

“Educational research that operates in a problem-posing rather than a problem-solving mode is, in this regard, not just research on or about or for education, but is, in a sense, itself a form of education as it tries to change mindsets and common perceptions, tries to expose hidden assumptions, and tries to engage in ongoing conversations about what is valuable and worthwhile in education and society more generally.” ( Biesta *et al*., 2019, p. 3)

We believe that for this type of education research to be more widespread, colleges of education, accordingly, need to complement the use of indirect indicators of scientific rigor with evidence of efforts to increase trustworthiness and usability. To foster such an approach, we ask those conducting, publishing, and assessing education research to consider doing the following:

1) Avoid simplistic models: Following the lead of the Open Science movement, DORA and others, all while considering complementing indirect measures of “impact” in assessment activities with more nuanced indicators of the quality, usability, and trustworthiness of a wide range of research products.
2) Avoid one-size-fits-all approaches: The usability of education research cannot be reduced to how practical or applied knowledge is, but to what extent it is potentially accessible to other researchers, stakeholders, and users ( *e.g.*, practitioners, policymakers, journalists, and the public).
3) Engage with field-specific models: Adopt and advocate for expanded indicators within systems of research assessment that are specifically relevant to other scholars in the field, as well as practitioners, policymakers, journalists, and the public.
4) Offer institutional support: The trustworthiness and usability of research need to be earned through interactive processes promoted and sustained institutionally. Individual scholars are trained to do good research and spend considerable effort in the analyses and syntheses of data, reviewing manuscripts, presenting at conferences, and the like. Making our results more usable and increasing trustworthiness requires time and effort in the form of producing complementary materials ( *e.g.*, podcasts, blogs, op-eds, video-commentaries, policy briefs, workshops). Colleges of education would greatly benefit and reduce some of the inequalities derived relatively simplistic models by allocating resources to increase the collective relevance of research production.
5) Account for context, language, and time: It is impossible to forecast the trajectory of scholarship, whereby the usability and trustworthiness of education research depends on the context of production, the languages used, as well as the time and timeliness of a publication.
6) Promote and reward efforts to remove barriers to research access and use: Recognize that quite a bit of very good scholarship is published in OA journals, as well as raise the awareness of the complexity of this model. Supporting OA publishing for researchers with limited resources, such as students, early career scholars, and scholars working in languages other than English, are also worthy ventures.

A first, though not an easy, move away from this unfair and ineffective system is to recognize alternatives and redirect our debates beyond the important, yet insufficient, question: How influential is the placement of a research contribution ( *e.g.*, article, book, or chapter) on the assessment of the merit of a scholar? Instead, we must embrace more comprehensive, and field specific systems of incentives and assessment oriented to the production of scholarship that contributes to the public good, encourages collaboration, and promotes interdisciplinary and intersectional research, and endeavors to increase access, trustworthiness, and responsiveness to both practical demands as well as conceptual challenges. As education researchers, our responsibility to avoid easy-to-implement models of scholarship assessment that end up producing more research that matters less.

## Data availability

There are no underlying data associated with this article.
