# Supplementary material for: Education research is still the hardest science: a proposal for improving its trustworthiness and usability
Source: F1000Res. 2022 Feb 24;11:230. [Version 1] doi: 10.12688/f1000research.109700.1 (PMC9294496; doi:10.12688/f1000research.109700.1)
Supplement: Peer review history

# Peer review: Reviewer response for version 1

**DOI:** 10.5256/f1000research.121238.r142161

**Published:** 2022-07-18

**Authors:** Daniëlle Verstegen

**Affiliations:** 1 Maastricht University, Maastricht, The Netherlands

This is an opinion article discussing that the current practice in measuring impact of educational research is inadequate. Although the authors are not the first to make this argument, they present an explicit analysis of why it is inadequate and also harmful. They include some example cases and refer elaborately to the literature.

 One argument that I would be inclined to add is that our current practice discourages research into topics or settings that need attention, but are less easy to publish.

 There is some overlap between the sections, but not very much.

 The authors end with a plea to focus more on trustworthiness and usability. That plea is convincing, but how we can judge trustworthiness and usability remains a bit vague.

 The manuscript ends with 6 guidelines for improvement. These are concrete, but  less nuanced than the text before and more clearly targeting Colleges of education (rather than the whole readership). For me, that decreases the trustworthiness and usability of this manuscript.

Is the topic of the opinion article discussed accurately in the context of the current literature?

Yes

Are arguments sufficiently supported by evidence from the published literature?

Yes

Are all factual statements correct and adequately supported by citations?

Yes

Are the conclusions drawn balanced and justified on the basis of the presented arguments?

Yes

Reviewer Expertise:

Instructional design, PBL, online/blended learning

I confirm that I have read this submission and believe that I have an appropriate level of expertise to confirm that it is of an acceptable scientific standard.

# Peer review: Reviewer response for version 1

**DOI:** 10.5256/f1000research.121238.r125407

**Published:** 2022-04-22

**Authors:** Jefferson Mainardes

**Affiliations:** 1 Department of Education, Universidade Estadual de Ponta Grossa, Paraná, Brazil

The article “Education research is still the hardest science: a proposal for improving trustworthiness and usability” discusses a highly relevant issue for the academic field: the emphasis given to metrics for the evaluation of academic performance. The article questions the one-size-fits-all assessment (for all areas) and suggests that academic impact should be analyzed from a wide range of research products, and not just a number of citations.

 A relevant aspect of the article is that, in addition to criticizing, it presents six proposals that could be used by faculties of education to complement conventional indicators. This point is highly relevant since the authors present contributions to minimize the existing problem.

 Some suggestions for corrections are the following:

1. on page 3 it is indicated that the JCR is generated by Scopus and JIF by the Web of Science. The correct information should be that the JCR is generated by Web of Science. It would be useful if other kinds of impact factor could be indicated (e.g. Scimago Journal Ranking – SJR, JIF, and so on).
2. The title of the article refers to the idea that research in education is still the hardest science. Some ideas from David C. Berliner (2002) are used. As it is mentioned in the title, readers could expect that this point would be more explored in the article. What are the authors’ arguments in relation to this assertion?  The complexity of educational research issues, educational inequalities in many parts of the world, the different onto-epistemological possibilities of theoretical foundations among other could be explored.

In general, this is a relevant article and should be accepted for indexing and wide dissemination, including the translation to other languages.

Is the topic of the opinion article discussed accurately in the context of the current literature?

Yes

Are arguments sufficiently supported by evidence from the published literature?

Yes

Are all factual statements correct and adequately supported by citations?

Yes

Are the conclusions drawn balanced and justified on the basis of the presented arguments?

Yes

Reviewer Expertise:

Education Policy, Research Ethics

I confirm that I have read this submission and believe that I have an appropriate level of expertise to confirm that it is of an acceptable scientific standard.
